# Development of Nucleic Acid Lateral Flow Immunoassay for Rapid and Accurate Detection of Chikungunya Virus in Indonesia

**DOI:** 10.4014/jmb.2108.08025

**Published:** 2021-09-30

**Authors:** Mandala Ajie, Dyshelly Nurkartika Pascapurnama, Susantina Prodjosoewojo, Shinta Kusumawardani, Hofiya Djauhari, Sukwan Handali, Bachti Alisjahbana, Lidya Chaidir

**Affiliations:** 1 Research Center for Care and Control of Infectious Disease (RC3ID), Faculty of Medicine, Universitas Padjadjaran, Bandung 40161, Indonesia; 2 Department of Internal Medicine, Faculty of Medicine, Universitas Padjadjaran/Hasan Sadikin Hospital, Bandung 40161, Indonesia; 3 Division of Parasitic Diseases and Malaria, Centers for Disease Control and Prevention, Atlanta, GA 30333, USA; 4 Department of Biomedical Sciences, Faculty of Medicine, Universitas Padjadjaran, Bandung 40161, Indonesia

**Keywords:** Chikungunya fever, NALFIA, clinical validation, open-source PCR, Indonesia

## Abstract

Chikungunya fever is an arboviral disease caused by the *Chikungunya* virus (CHIKV). The disease has similar clinical manifestations with other acute febrile illnesses which complicates differential diagnosis in low-resource settings. We aimed to develop a rapid test for CHIKV detection based on the nucleic acid lateral flow immunoassay technology. The system consists of a primer set that recognizes the E1 region of the CHIKV genome and test strips in an enclosed cassette which are used to detect amplicons labeled with FITC/biotin. Amplification of the viral genome was done using open-source PCR, a low-cost open-source thermal cycler. Assay performance was evaluated using a panel of RNA isolated from patients&rsquo; blood with confirmed CHIKV (*n* = 8) and dengue virus (*n* = 20) infection. The open-source PCR-NALFIA platform had a limit of detection of 10 RNA copies/ml. The assay had a sensitivity and specificity of 100% (95% CI: 67.56% - 100%) and 100% (95% CI: 83.89% - 100%), respectively, compared to reference standards of any positive virus culture on C6/36 cell lines and/or qRT-PCR. Further evaluation of its performance using a larger sample size may provide important data to extend its usefulness, especially its utilization in the peripheral healthcare facilities with scarce resources and outbreak situations.

## Introduction

Chikungunya fever is a mosquito-borne viral disease caused by the *Chikungunya* virus (CHIKV). First described during the outbreak in Tanzania in 1952, it is known to be transmitted by infected *Aedes* mosquitoes [1]. CHIKV is an RNA virus that belongs to the *Alphavirus* genus of the family *Togaviridae*. Some distinctive symptoms of CHIKV infection are the immediate onset of high-grade fever along with joint pain which lasts for a few days or might be extended to weeks. Other common symptoms also include myalgia, nausea, exhaustion, muscle pain, and skin rash [2].

Various methods are used to diagnose CHIKV infection [3]. Viral culture is considered to be the gold standard for the diagnosis but requires highly skilled trained laborants [4, 5]. qRT-PCR offers a sensitive and relatively rapid diagnosis but is not suitable for application in peripheral healthcare facilities as it needs to be operated and interpreted by trained technicians [6-9]. Antibody testing, which detects the presence of the host’s IgM and/or IgG against CHIKV, is available in a rapid test form and thus can be used without specialized training. However, antibodies are only detectable 5-7 days after the onset of the illness, making it unsuitable for detecting the infection during its acute phase [10]. Moreover, the sensitivity of the rapid tests, which varies between 20-95%, is far inferior to other diagnostic methods [8-11].

In addition to the limitations of the CHIKV diagnostic methods, the clinical similarity of CHIKV to other arboviral infections challenges the diagnosis based on clinical symptoms alone. Some arboviruses within the same geographical regions or vector species such as dengue virus (DENV) and Zika virus infection might show similar symptoms and leads to a false diagnosis of Chikungunya fever [10-16]. Due to access limitation and other challenges for CHIKV diagnosis, finding alternative options for CHIKV testing is urgently needed. Such alternatives must be applicable at the point of care and/or elsewhere in access- and resource-limited settings, where CHIKV outbreaks primarily occur.

In this study, we developed a molecular-based rapid diagnostic assay for detecting CHIKV. Detection is based on a nucleic acid lateral flow immunoassay (NALFIA) system which utilizes a strip in a housed cassette that recognizes protein tags that are attached to the pathogenic genome fragment through PCR amplification [17-19]. Replacing electrophoresis or qRT-PCR with the NALFIA system allows the assay to be performed more easily. We combined the NALFIA system with an open-source thermal cycler to make the test easier to transport and available in limited settings. This innovative system is expected to facilitate the early detection of acute phase CHIKV infection.

## Materials and Methods

### Sample Selection

The assay was tested on blood sera with known viral acute febrile illness retrieved from the clinical specimen collection of Universitas Padjadjaran’s Infectious Disease Research Center. Samples were collected consecutively from individuals with acute febrile illness from July 2014 to March 2016. The etiology of the fever was identified before this study [20]. Determination of CHIKV infection was done using a composite reference standard consisting of virus culture on C6/36 cell line and qRT (real-time quantitative reverse transcription)-PCR on the *nSP* gene; samples were considered to be CHIKV (+) when they were culture- and/or qRT-PCR- positive for CHIKV. Details on virus culture and qRT-PCR protocols can be found in our previous studies [20, 21]. Blood sera from patients with known viral infections were retrieved and viral RNA was extracted using the QiaAmp Viral RNA Mini Kit (Qiagen, Germany). The resulting RNA extracts were stored at -80°C before analysis. The protocol of this study was approved by the Ethics Committee of Hasan Sadikin Hospital (LB.02.01/C02/515/1/2015; LB.02.01/C02/2352/II/2016.

### Primer Design

Conserved regions from local CHIKV strains were identified by analyzing 62 CHIKV genome sequences from Indonesia using BioEdit’s multiple alignments. The conserved regions were processed using Primer3Plus (Free Software Foundation, USA) to generate a set of primers targeting 161 bp of the E1 region (CHIKE1-F: 5’-TAAGCTCCGCGTCCTTTACC-3’; CHIKE1-R: 5’-AGACGTCGCCTTTGTACACC-3’. The 5’ ends of both CHIKE1-F and CHIKE1-R were labeled with FITC and biotin, respectively, for NALFIA visualization.

**Fig. 1:**
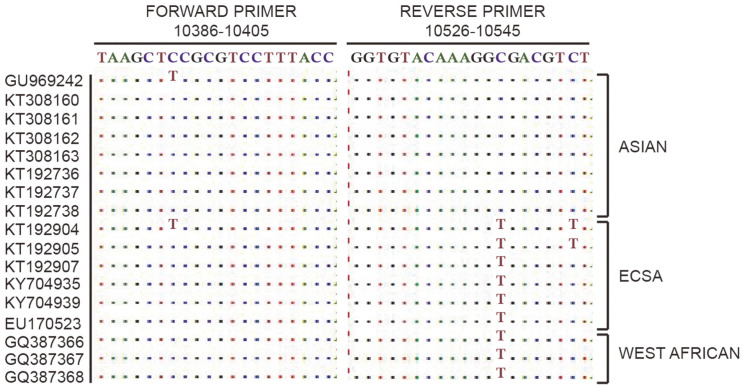
Sequence alignment between primers set with E1 sequences from CHIKV.

Possible variations in the chosen region were analyzed by aligning the primer set with 17 CHIKV isolates from different genotypes (Fig. 1).

### Conventional RT-PCR

One-step RT (reverse-transcription)-PCR was done using the One-Step RT-PCR Kit (Qiagen). The reaction was run on 25 μl mixture containing 0.5 μM of CHIKE1-F primer, 0.6 μM of CHIKE1-R primer, 5 μl of RNA extracts, and Qiagen One-Step RT-PCR reagents under the condition detailed below (Table 1). Unlike qRT-PCR, which uses fluorescence to detect cDNA, in conventional one-step RT-PCR, amplicons are visualized using electrophoresis on 2% agarose gel. Each batch of sample testing was done in a blinded manner. The quality of the testing was stringently controlled using positive control from the RNA isolated from CHIKV culture and RNase-free water as a negative reaction control.

The assay was done in duplicate using the open-source thermal cycler design from Chai Inc. (USA). OpenPCR is a low-cost, open-source thermal cycler. The machine comes in 5 different parts (heated lid, laser-cut case, machine ‘brain’, machine ‘core, power supply,’ and heat sink) that need to be assembled into a working thermal cycler. The system has an average ramp rate of 1°C/s with a temperature accuracy of 0.5°C and well-to-well temperature uniformity of 0.3°C. Details on the specifications of the machine and the build instructions can be found on the manufacturer’s website [22]. The performance of the assay carried out using the open-source PCR system was compared to the ones done using a conventional thermal cycler, *i.e.*, commercial thermal cycler from Bio-Rad (USA).

### Nucleic Acid Lateral Flow Immunoassay (NALFIA)

NALFIA was done using the commercialized NALFIA cassette according to the manufacturer’s protocol. Five microliters of PCR products from reactions conducted on open-source PCR was mixed with 70 μl of the assay extraction buffer. The mixture was then added to the test cassette’s sample well, resulting in the diffusion of the solution through the test strip. The test result was read within a window of 10 min; the appearance of a line on both the test line and control line indicated a positive result, whereas a line appearing on the control line alone indicated a negative result. Tests in which the control line failed to develop were deemed invalid and a retesting was performed. All changes that occurred after the 10 min incubation period were deemed invalid and ignored.

### Feasibility Test of NALFIA Development

To test the feasibility of visualizing amplicons on the NALFIA cassette, a preliminary test using labeled primers was conducted. RNA extracted from CHIKV culture (10^8^ RNA copies/ml) and the negative reaction control of RNase-free water template were subjected to PCR conditions as previously described. PCR products were then visualized on the test cassette and electrophoresis.

### Performance Evaluation

The performance of the open-source PCR-NALFIA system was assessed in terms of its least limit of detection, sensitivity, and specificity. The least limit of detection was determined using CHIKV genomic RNA stock (10^8^ RNA copies/ml) performed until the RNA stock reached the concentration of 10^0^ RNA copies/ml and the assay was performed on each of the dilutions. The least limit of detection was defined at the lowest RNA concentration where a band could be detected in the test cassette. Amplicon detection using gel electrophoresis was used as a control for the assay.

The sensitivity and the specificity of the assay were determined by comparing the test results with a composite reference standard consisting of virus culture on the C6/36 cell line and qPCR assay that was done before this study. Concordance between open-source PCR-NALFIA and other diagnostic methods was measured using Cohen’s Kappa analysis. All the statistical analyses were performed in SPSS 24 for Windows.

## Results

### Sample Selection

A diagnosis could be proven on 190 of 600 samples screened in a fever diagnostic study (23). Viral infections occurred in 89/190 samples, with the rest confirmed as bacterial infections (94/190), malaria (4/190), and co-infections of DENV and salmonella (3/190). Of all proven viral infection cases, 8/89 cases were CHIKV infection, whilst the remaining cases were DENV infection. All CHIKV-positive samples and 20/81 DENV-positive samples were retrieved for evaluating the assay’s performance [23].

### NALFIA System Development

**Fig. 2:**
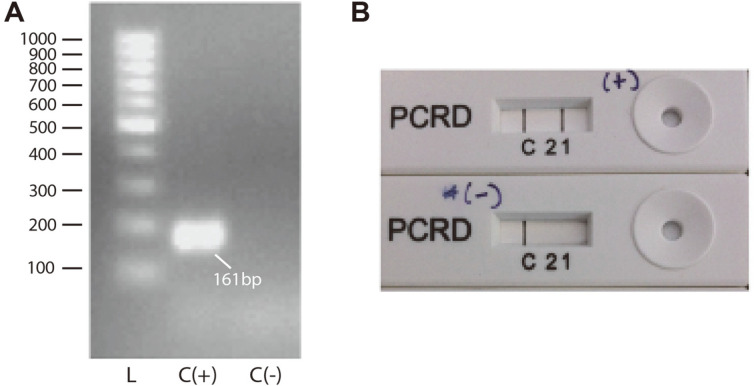
A) electrophoresis results on defined controls. Lane L: ladder 100 bp, lane 2: C+ (positive control), lane 3: C- (negative control). B) NALFIA results in defined controls. From top to bottom: positive control, negative control. CHIKV RNA extract with initial concentration of 108 copies/ml was used as the positive control in the tests.

A clear band was developed on a positive control line on the test strip within 10 min of incubation whilst no visible band could be observed on the negative control line (Fig.2). Small-sized (<100bp) nonspecific amplification products could be seen on the electrophoresis photo, both in the positive and negative control. However, those products did not affect visualization on the test cassette, suggesting that the products did not form from interactions between FITC and biotin-labeled primers.

### Least Limit of Detection

**Fig. 3:**
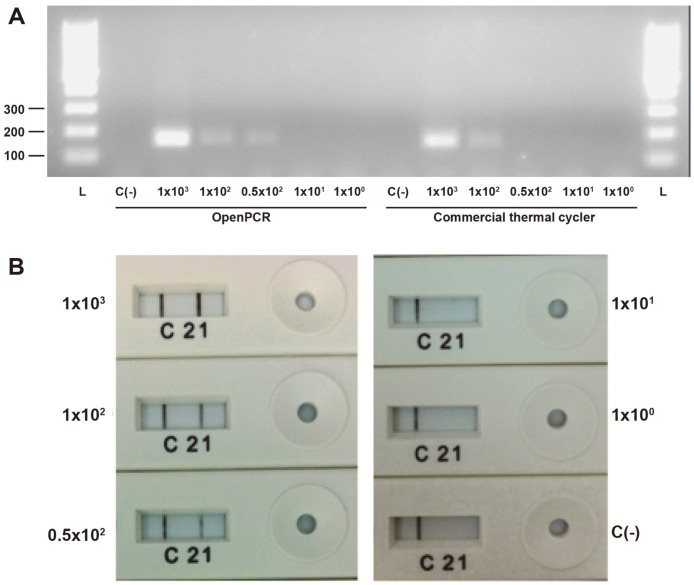
A) electrophoresis results on serially-diluted CHIKV RNA amplified using openPCR system and commercial thermal cycler. B) NALFIA visualization of CHIKV RNA amplicons generated with openPCR. Concentrations are stated in RNA copies/ml format. L: 100 bp ladder, C(-) negative control.

The limit detection of the assay was assessed on three different platforms: 1) commercial thermal cycler with electrophoresis visualization, 2) conventional PCR on open-source PCR system with electrophoresis visualization, and 3) conventional PCR on open-source PCR system with NALFIA visualization (Fig. 3). CHIKV RNA stock, serially diluted to 10^0^ CHIKV RNA copies/ml was used as the templates in the assessment.

The least limit of detection was defined by the presence of an observable band in the reaction with the smallest concentration of template (Table 2).

The least limit of detection for the open-source PCR-NALFIA platform was found to be the lowest (10 CHIKV RNA copies/ml) compared to the commercial thermal cycler-electrophoresis (100 CHIKV RNA copies/ml) and open-source thermal cycler-electrophoresis (50 CHIKV RNA copies/ml. These results showed that NALFIA could outperform electrophoresis in detecting PCR amplicons with lower concentrations.

### Diagnostic Performance on Clinical Samples

The open-source thermal cycler-NALFIA system showed good accuracy in determining the presence of CHIKV infection in clinical specimens. The sensitivity and specificity of the assay were 100% (95% CI: 67.56% - 100%) and 100% (95% CI: 83.89% -100%), respectively, compared to the composite reference standard previously used in confirming the diagnosis. The agreement between the open-source PCR-NALFIA system with other diagnostic tests used in forming the composite reference standard was assessed using Cohen’s Kappa analysis (Table 3).

The open-source PCR-NALFIA system showed moderate concordance with virus culture on the C6/36 cell line (K=0.7; 95% CI: 0.40-1.00). On the other hand, the open-source PCR-NALFIA system had high concordance with qRT-PCR (K=0.91; 95% CI: 0.73-1.00), successfully detecting one CHIKV-positive sample that was deemed negative by qRT-PCR. The open-source PCR-NALFIA system also showed high concordance with the same assay performed on the open-source PCR-electrophoresis system (K=0.91 95% CI 0.73, 1.00), with the difference caused by the lower least limit detection of the open-source PCR-NALFIA system.

## Discussion

In this study, we developed a nucleic acid lateral flow immunoassay (NALFIA) system for the detection of Chikungunya virus (CHIKV) infection. We then compared the performance of the assay when used with two different thermal cyclers, a commercial thermal cycler, and a self-assembled open-source thermal cycler, and found the least limit of detection of open-source PCR-NALFIA to be lower than both open-source PCR-electrophoresis and commercial thermal cycler-electrophoresis. Assessment of the assay’s accuracy using clinical samples showed that the open-source PCR-NALFIA system had excellent sensitivity and specificity compared to the composite reference standards. The assay had a moderate agreement with virus culture (Cohen’s Kappa 0.70; 95%CI: 0.40-1.00) and high agreement with both conventional and quantitative PCR (Cohen’s Kappa 0.91; 95%CI 0.73-1.00).

It is interesting to note that the assay performed using the open-source PCR system had a better least limit of detection compared to the commercial thermal cycler. The important parameters that determine the reliability of a thermal cycler are its ramp rate (the rate of temperature increase and decrease per second), accuracy (the ability of the machine to hit and maintain the determined temperature), and well-to-well variations (the temperature difference between different wells) [24, 25]. The open-source PCR system and the commercial thermal cycler have a comparable range of temperature accuracy and well-to-well variations but the commercial thermal cycler system has a higher ramp rate (1°C/s vs 3.3°C/s), which should have made the commercial thermal cycler system a slightly better instrument by comparison [22, 26]. Thus, differences in the performance between two machines might be caused by factors other than their inherent specifications, such as manufacture date and frequency of use. This finding highlights the importance of regular check-ups and maintenance for diagnostic instruments.

The validation of the assay’s performance on clinical samples showed that the open-source PCR-NALFIA system has excellent sensitivity and specificity compared to our composite reference standard. Our assay could identify the presence of the CHIKV genome in samples that were 1) culture-positive, qRT-PCR-negative, 2) culture-positive, qRT-PCR-positive, and 3) culture-negative, qRT-PCR-positive. The assay had a moderate agreement with virus culture and high agreement with other nucleic acid-based assays. This result was to be expected as virus culture, despite having high specificity, had pooled sensitivity of approximately 40.5% [3]. On the other hand, the assay had a high Cohen’s Kappa value with nucleic acid-based assay and could even successfully detect one CHIKV-positive sample that was culture-positive, qRT-PCR-negative. The difference might be attributed to the different targets used in the assays; the open-source PCR-NALFIA system amplified *E1* fragment whilst the qRT-PCR assay we previously used amplified *nSP* [20, 21]. The results showed that the open-source PCR-NALFIA system performed well in detecting the presence of CHIKV in clinical samples. These numbers, however, need to be interpreted with caution as the evaluations were performed on a small number of samples. A study with a larger sample size should be performed to better assess the accuracy of the assay.

Compared to regular PCR-based assays, our system requires less investment in diagnostic infrastructures. OpenPCR machine, which costs ~$500 per unit (https://shop.tori.st/), is much cheaper than commercial thermal cyclers as well as the qPCR system, thereby making it more accessible to peripheral diagnostic centers with limited resources. The utilization of NALFIA further reduces the need of equipment for visualization, *i.e.*, electrophoresis cells and transluminator needed in conventional PCR system, and fluorimeter in qPCR system. Furthermore, visualization using NALFIA is practical and quick, taking fifteen minutes to be completed, as opposed to electrophoresis, which can take more than an hour. This makes the running speed of the whole OpenPCR-NALFIA system comparable to qRT-PCR assays. The assay indeed takes a longer time than the rapid diagnostic kits for CHIKV. However, to this day, all rapid immunochromatographic assays for CHIKV rely on either antigen or serological detection, with sensitivity varying between 20-95% [4, 27-29]. Serological testing for CHIKV has also been shown to be less sensitive during the acute phase of the infection [3, 4, 10].

In conclusion, we have developed a nucleic acid-based diagnostic system for CHIKV infection with excellent sensitivity and specificity. Our open-source PCR-NALFIA system offers sensitive and specific nucleic acid-based testing for CHIKV that can be used for early detection of CHIKV. Moreover, as we utilize a cheap, open-source thermal cycler as well as an easy-to-use test cassette, the assay can potentially be used in peripheral healthcare facilities with scarce resources.

## Figures and Tables

**Table 1:** PCR conditions.

Step	No. of cycles	Temp. (C)	Duration (mm:ss)
Reverse transcription	1	50	30:00
TaqPol activation	1	95	15:00
Amplification	35	94	00:30
		55	00:30
		72	01:00
Final elongation	1	72	10:00

**Table 2:** Least limit of detection of CHIKV assay on different platforms. Band development intensity was rated in (+) to (+++) scale, with (+) being the least intense.

RNA copies/ml	Commercial thermal cycler	Open-source PCR	

		Electrophoresis	NALFIA cassette
1 × 10^3^	+++	+++	+++
1 × 10^2^	++	++	++
0.5 × 10^2^	-	++	++
1 × 10^1^	-	-	+
1 × 10^0^	-	-	-
C-negative	-	-	-

**Table 3:** Agreement between open-source PCR-NALFIA system and other assays.

Method		NALFIA cassette status		Kappa (95% CI)

		Positive (+)	Negative (-)	
Viral culture	Positive	5	0	0.70 (0.40-1.00)
	Negative	3	20	
qRT PCR	Positive	7	0	0.91 (0.73-1.00)
	Negative	1	20	
Conventional RT-PCR	Positive	7	0	0.91 (0.73-1.00)
	Negative	1	20	
